# Efficacy of Serial Ultrasonographic Examinations in Predicting Return to Play in Agility Dogs with Shoulder Lameness

**DOI:** 10.3390/ani12010078

**Published:** 2021-12-30

**Authors:** Maria Grazia Entani, Alessio Franini, Ludovica Dragone, Gabriele Barella, Fabio De Rensis, Giliola Spattini

**Affiliations:** 1 Sporty Dog, 25123 Brescia, Italy; mariagrazia.entani@sportydog.vet (M.G.E.); franinivet@gmail.com (A.F.); 2 Dog Fitness, 42124 Reggio Emilia, Italy; ludovica@dogfitness.it; 3 Clinica Veterinaria CMV, 21100 Varese, Italy; gabriele.barella@gmail.com; 4 Department of Veterinary Medical Science, Parma University, 43126 Parma, Italy; fabio.derensis@gmail.com; 5 Clinica Veterinaria Castellarano, 42014 Castellarano, Italy

**Keywords:** ultrasound, shoulder injury, agility dogs, physiotherapy, return to play

## Abstract

**Simple Summary:**

Agility dog is one of the most popular canine sports among pet owners, and agility-related soft tissue injuries are becoming frequent in dogs. The need to check the progression of shoulder tendon healing in order to permit a safe return to sport is now urgent. The aim of the present study was to help veterinary sports medicine professionals check the progression of shoulder tendon healing in lesions that have been treated with a standardised physiotherapeutic program. The results showed that ultrasonographic examination could be considered a reliable method of following the progression of healing of shoulder tendon lesions when carried out every two months within the six months after injury, together with the administration of a standardised physiotherapeutic program. The ultrasonographic protocol should be associated with a clinical evaluation in order to give the correct return to play time to the owners.

**Abstract:**

The aim of this study is to investigate the use of shoulder ultrasound as a method of predicting the likelihood of returning to competition in agility dogs with shoulder teno-muscular injuries after a standardised rehabilitation protocol. Thirty-two agility dogs with a clinical and ultrasonographic diagnosis of shoulder teno-muscular injury were included in a prospective study with physical and ultrasound examinations at the time of diagnosis (T0) and at two (T2), four (T4) and six (T6) months; during this period, the dogs received rehabilitation treatments. The endpoint of the study was to obtain information regarding participation in agility competitions 12 months after diagnosis, based on telephone interviews with the owners. The clinical lameness score (CLS) and the ultrasound lesion score (ULS) were used as outcome measurements. The CLS indicated partial recovery from a shoulder injury at T2 (78%), while the ULS indicated no satisfactory recovery at T2 in any patient. At 4 months, the CLS alone was not a valuable predictor of full recovery from a shoulder injury in agility dogs. Relative Risk indicated that, at T2, ultrasound was 23.8 times more valuable in identifying a shoulder lesion as compared to clinical lameness score (CLS), and it was 2.53 times more valuable at T4.

## 1. Introduction

Agility dog has increased its popularity in recent years, and it is a worldwide performed canine sport. Competition has become more challenging for dogs, and injuries have increased in number and severity. Before 2009, reports of agility-related injuries were anecdotal [1]. From 2009 to 2018, preliminary retrospective survey-based studies reported that soft tissue injuries were predominant and that the shoulder, back, phalanges and neck were the most commonly affected regions [2,3].

Ultrasonographic evaluation of the canine shoulder was first described in the late 1990s. Due to its availability, relatively low cost and ease of use without patient sedation, ultrasound (US) has become a fundamental diagnostic tool for investigating shoulder teno-muscular diseases in dogs [4].

Despite the lack of scientific evidence, physiotherapy is becoming the dominant tool for the non-surgical management of sports-related soft tissue injuries in dogs. However, the physical treatment options for these lesions have never been tested, and the choice of the time to return to sports is mainly based on therapist experience [5]. Only two papers were published regarding the return to play (RTP) of agility dogs after an orthopaedic injury [6,7]. No studies have been published to assess the efficacy of a standardised physiotherapeutic program in veterinary medicine. The aim of this study was to propose a model for serial ultrasonographic evaluations of agility-related shoulder injuries during a standardised rehabilitation protocol of six months duration.

## 2. Materials and Methods

### 2.1. Dogs

From May 2014 to May 2018, 32 agility dogs performing in agility competitions, which were presented to Clinica Veterinaria Castellarano (Italy) for a US examination of the shoulders, were included in the study. A consensus statement was signed by the owners when they agreed to enrol the dogs in the trial.

The inclusion criteria were: at least one month of persistent forelimb lameness due to trauma that occurred during agility training or performance, a clinical orthopaedic evaluation suggesting shoulder pain, a radiographic study of the shoulders and elbows ruling out major orthopaedic diseases, ultrasound diagnosis of shoulder teno-muscular diseases with three ultrasonographic follow-ups within six months, enrolment in a rehabilitation program in a specialised canine sports medicine clinic and telephone follow up six months after resuming competition.

### 2.2. Clinical Evaluation

All patients were evaluated by experienced orthopaedic surgeons. A worldwide clinically used 0–4 grading system was applied to categorise the lameness (Clinical Lameness Score (CLS]) (Table 1). The patients with 4th-degree lameness were excluded because they required pharmacological assistance, which could have interfered with the results of the physical therapy.

### 2.3. Ultrasound Evaluation

The patients were positioned in lateral recumbency with the examined shoulder upward; the shoulder area was clipped, and ultrasound gel was applied. All the ultrasonographic examinations were carried out by the same operator (GS) using an 8–18 MHz hockey stick linear probe and run by a GE LOGIQ S8* ultrasound machine (GE Healthcare Italian Division. GE LOGIQ S8 General Imaging Ultrasound). The examination, including both shoulders, was carried out at time 0, using a standardised protocol [8]. The same protocol was repeated at two, four and six months after diagnosis [9,10]. The two-month interval between evaluations was chosen according to the healing process of muscle and tendons described in human and equine literature, allowing the ultrasonographic visualisation of recovery changes [11,12]. At the end of the remodeling phase, the authors avoided an extra ultrasonographic examination 12 months after T0. It was predicted, according to the literature, that the ultrasonographic changes would be minimal. At the same time, the function of the repaired tendon or muscle would be different. The phone interview with the owner assessed this phase. A grading system generated by the authors, the Ultrasound Lesion Score (ULS), was adopted for staging the ultrasound lesions and is reported in Table 2.

### 2.4. Physiotherapeutic Protocol

The physiotherapeutic protocol was based on the literature available and the experience of the authors [4,13]. From time zero to two months, the rehabilitation sessions were carried out using physical and instrumental modalities. The frequency of the treatments (once or twice weekly) depended on the ultrasonographic findings. Controlled and gentle Passive Range Of Motion (PROM) exercises were performed and were associated with proprioceptive exercises [14,15,16,17]. The affected area was clipped, and a coupling medium was applied when needed for diathermy (Fisioline Radiant^®^ Veterinary) and Therapeutic Ultrasound (TUS, Lumix^®^ CPS Veterinary Laser). Diathermy in low-level energy mode (20 or 25% energy level for 15 min of treatment) was the first modality to be applied, followed by TUS at 3.3 MHz in pulsed mode (20%) and 0.5–0.7 W/cm^2^. Laser Therapy was used at 3–9 J/cm^2^ in scanning mode (Fisiosonic^®^ Veterinary). Approximately fifteen days after the beginning of instrumental and manual therapy, after checking the clinical condition of the patient, Underwater Treadmill (UWT) exercise was introduced at the end of the instrumental session (Hydro Physio^®^ TM). The water level was up to the great trochanter to reduce the weight-bearing stress to 38% of the load [18]. The speed of the treadmill and the length of the hydrotherapy session, which never exceeded fifteen minutes, varied as a function of the clinical parameters of the patient (age, weight-bearing and muscular conditioning). Exercise at home was restricted, and jumping, running, sudden movements and stairs had to be avoided. Short leash walks of a maximum of 15 min on a smooth sidewalk were allowed frequently during the day (4/5 times per day).

From two to four months, depending on the US follow-up results, the therapeutic exercises were continued and were gradually intensified at home, with the short leash walk increased to up to 25 min.

The four-six month rehabilitation period, based on the US report, was divided into two parts. In the first two weeks, the once-per-week rehabilitation session was continued, with a reduction in the instrumental component, gradually removing TUS but continuing diathermy. Gentle PROM and proprioceptive exercises were maintained, and UWT was intensified with a counter-current walk. A longer walk, of a maximum of forty minutes, was allowed at home but always avoiding sudden movements, running and stairs. In the second part, if no signs of discomfort were evident after increased activity, sport exercises were gradually introduced with shorter but more intense rehabilitation workload sessions. Instrumental procedures were completely stopped, and sports-related training was started.

### 2.5. Statistics

Fisher’s exact test was used to evaluate the differences in the CLS and the ULS at the first visit (T0), at two months (T2), at four months (T4) and at six months (T6). *p* < 0.05 was considered to be statistically significant.

Relative Risk (RR) was measured at T2, T4 and T6, considering the ULS as the experimental group and the CLS as the control group. *p* < 0.05 was considered to be statistically significant [19].

## 3. Results

Fourteen Border Collies, nine Australian Shepherds, four Shetland Sheepdogs and five Mixed Breeds were selected. The age ranged from two years and four months to eleven years and one month (mean 5.7 years). Patient data and relevant lesions are summarised in Supplementary Materials Table S1.

**Figure 1:**
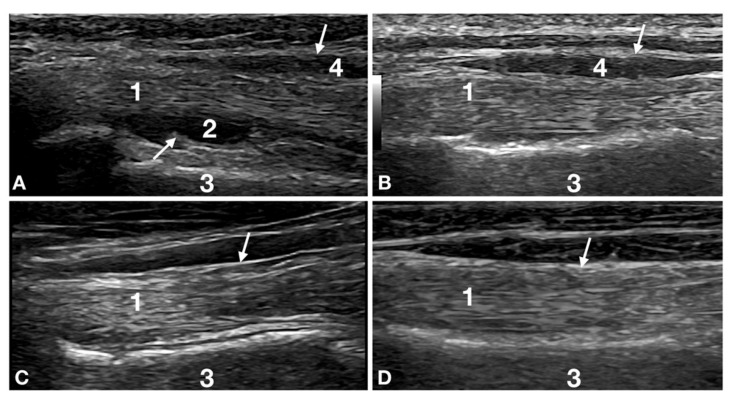
Images of the right biceps tendon distal to the bicipital groove of patient number 12 at Time 0, Time 2, Time 4 and Time 6 are reported. The image shows the progression of healing. (**A**) Time 0: biceps tendon (1) is not uniform in diameter with a poor fibrillar pattern. The tendon is less echoic with fewer, less defined, thin, hyperechoic lines running along its longitudinal axis. Shoulder effusion (2) is seen between the deeper interface of the tendon and the cranial humeral surface (3). Tendon sheath thickening (arrows) and effusion (4) are evident. (**B**) Time 2: the biceps tendon (1) is more uniform in diameter thickness but is not the same as the contralateral (not shown but similar to panel d). The fibrillar pattern is mildly increased, and the tendon is more homogeneous and echoic. The shoulder effusion has resolved. The superficial tendon sheath (arrow) is still moderately thickened with mildly reduced effusion (4). (**C**) Time 4: the biceps tendon (1) is more echoic and is uniform in thickness, with a diameter comparable with the contralateral limb. The fibrillar pattern is better defined but still not identical to the contralateral tendon (compared with panel d). The superficial tendon sheath (arrow) is back to normal for thickness, and there is no visible effusion. (**D**) Time 6: the biceps tendon (1) is echoic with a fine fibrillar pattern, typical of tendons. The echotexture, echogenicity and thickness are identical to the contralateral limbs (not shown). The superficial tendon sheath (arrow) is back to normal for thickness, and there is no visible effusion. All the lesions are resolved, and no degenerative changes are seen in this patient.

At T0, the most frequent lesion was a reduction in the biceps tendon diameter (24/32; 75%). In general, this finding was associated with fluid accumulation in the biceps tendon sheath (17/32; 53%), shoulder joint effusion (12/32; 37%) and tendon sheath thickening (11/12; 92%) (Figure 1).

**Figure 2:**
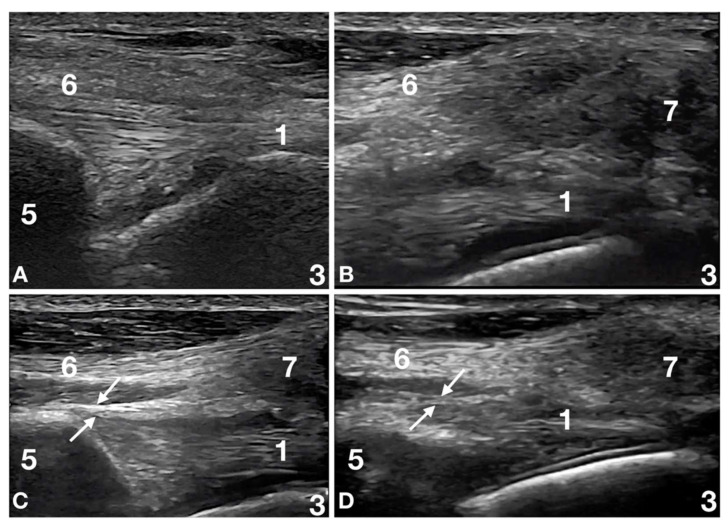
Images of the left shoulder at the level of the biceps tendon origin and supraspinatus humeral insertion of patient number 14 at Time 0, Time 2, Time 4 and Time 6. (**A**) Time 0: the supraglenoid tubercle (5) with the origin of the biceps tendon (1) is seen. There is no clear definition between the biceps tendon (1), the joint capsule (between arrows in panels c and d), and the tendinous (6) and fibro-cartilaginous (7) (visible in panel b, c and d) portions of the supraspinatus tendon. At dynamic examination, there were no relative motions between the biceps and the supraspinatus tendons. The joint capsule was not identified between these two structures. This condition is called adhesive capsulitis [20]. (**B**) Time 2: There is a better definition between the biceps tendon (1) and the tendinous (6) and fibrocartilaginous (7) portions of the supraspinatus tendon; however, these structures show adhesions at dynamic examination. The joint capsule (between the arrows in panels **C**,**D**) is not identified. The adhesive capsulitis is still present. (**C**) Time 4: There is a better definition between the biceps tendon (1) and the tendinous (6) and fibrocartilaginous (7) portions of the supraspinatus tendon, and no adhesion is seen at dynamic examination. The joint capsule (between the arrows) is thickened and irregular but is identified as an independent structure. The adhesive capsulitis is resolved. (**D**) Time 6: the biceps tendon (1) is clearly defined with a richer fibrillar pattern. The joint capsule (between the arrows) is better defined, less thickened and more regular than before. No interference is seen at dynamic examination between the joint capsule and the biceps tendon (1). No interference is visible between the joint capsule and the tendinous (6) and fibrocartilaginous (7) portions of the supraspinatus tendon.

Thirteen patients (13/32; 41%) had biceps tendon lesions associated with supraspinatus tendon lesions, and adhesions between those two structures were present in seven of the thirteen dogs (7/13; 54%) (Figure 2).

**Figure 3:**
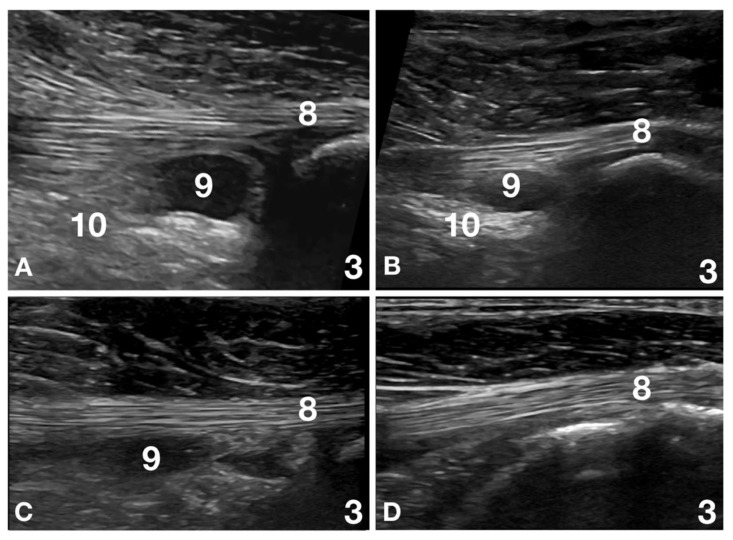
Images of the left shoulder at the level of the infraspinatus muscle and tendon insertion of patient number 18 at Time 0, Time 2, Time 4 and Time 6 are reported. (**A**) Time 0: the infraspinatus tendon (8) is thin and relatively ill-defined at its humeral insertion (3). A large anechoic area (9) surrounded by scattered artefacts (10) is seen in the region of the teno-muscular junction. This lesion was considered a muscle rupture and was differentiated by an enlarged infraspinatus bursa due to the size, location and scattered artefacts associated with acute inflammation. The severe pain at palpation of the region was another element that helped to differentiate the pathology from normal anatomy. (**B**) Time 2: the infraspinatus tendon (8) is thin as compared with the contralateral limb but is better defined with a more pronounced fibrillar pattern. The anechoic area (9) is reduced in size, and the associated scattered artefacts (10) are also reduced. Mild pain was still present on palpation. (**C**) Time 4: the infraspinatus tendon (8) has nearly the same diameter as the contralateral limb. The fibrillar pattern is compact and shows good alignment. The anechoic area (9) is reduced in size, more difficult to see, lacks well-defined margins, and is not associated with scattered artefacts. No pain was present on palpation. (**D**) Time 6: the infraspinatus tendon (8) shows the same diameter and fibrillar pattern as the contralateral limb. The anechoic area (9) is no longer visible and is not associated with scattered artefacts. No significant differences with the contralateral limb are visible. No pain was present on palpation.

In one patient, adhesion of the joint capsule with an altered supraspinatus tendon was seen, not associated with changes in the adjacent biceps tendon. In three patients (3/32; 9%), including those previously mentioned, an isolated supraspinatus lesion, considered clinically relevant, was the most important finding. In four patients (4/32; 12%), the most relevant finding was a partial rupture and thinning of the infraspinatus tendon and, in two patients (2/32; 6%), and an associated muscle rupture was present (Figure 3).

**Figure 4:**
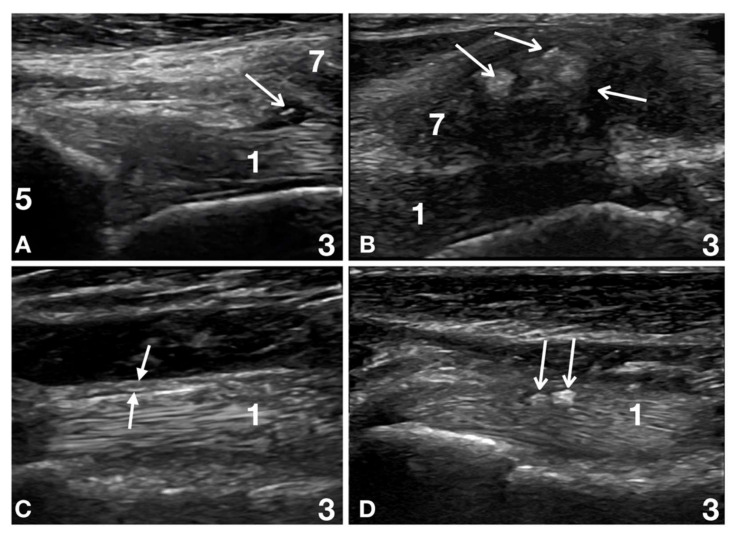
In some of the degenerative lesions, not considered clinically relevant but still present at Time 6, ultrasonographic evaluation is shown. (**A**) Patient 27: right shoulder at the level of the biceps tendon (1) supraglenoid origin (5) at Time 6. A well-defined, fibre-rich, uniform in diameter biceps tendon (1) is effacing a focal dilatation of the tendon sheath, which presents a plaque-like, thin, mineralised lesion (empty arrowhead). At the dynamic examination, the plaque-like mineralised structure was attached to the tendon sheath, and the limited effusion prevented friction with the biceps tendon. The patient was competing with no issues six months after the end of the physical therapy. (**B**) Patient number 3: left shoulder at Time 6. Several mineralised lesions (empty arrowhead) create a clean acoustic shadow and partially hide the biceps tendon underneath. These lesions were present at the first presentation (not the only lesions present) and did not change between examinations, despite the different clinical signs of the patient over time. As reported in the literature, mineralised lesions in the supraspinatus tendon are often not considered to be clinically significant unless they are on the surface of the tendon and interfere with the movements of the joint capsule and the biceps tendon underneath. (**C**) Patient number 15: left biceps tendon (1) distal to the bicipital groove, Time 6. There is a uniformly thickened tendon sheath (between the full arrowheads) in this patient. The dog was performing well six months after the end of physiotherapy. The smooth surface and lack of associated tendon sheath effusion are relevant factors in defining this thickness not to be clinically relevant. The tendon sheath may be back to its original thickness over time. (**D**) Patient number 9: left biceps tendon (1) distal to the bicipital groove, Time 6. A well-defined, fibre-rich, uniform biceps tendon (1) is adjacent to two large, rounded, hyperechoic structures (empty arrowheads). At the dynamic examination, the rounded, most likely partially mineralised (acoustic shadowing was partially present) were attached to the tendon sheath and were sliding, apparently not causing friction with the biceps tendon. The patient was performing well six months after the end of the physical therapy.

In five patients (5/32; 16%), associated infraspinatus and biceps tendon lesions were detected. Four of these five (4/5; 80%) showed an associated supraspinatus lesion, suggestive of a severe instability shoulder syndrome. Muscle ruptures were observed as the only lesion in one patient (1/32; 3%); they were present in two patients (2/32; 6%) associated with an infraspinatus tendon lesion and in one patient (1/32; 3%) with biceps tendon diameter reduction. Minimal, chronic, degenerative changes considered equivocally clinically significant were seen in a few patients. They included a thickened biceps tendon sheath (3/32; 9%), a mineralised biceps tendon sheath (3/32; 9%), and mineralised areas in the supraspinatus tendon (2/32; 6%) (Figure 4).

The statistical results are summarised in Table 3 and Table 4.

At T0, all the patients had 1st-, 2nd- or 3rd-degree lameness in one or both front limbs. Significant differences (*p* < 0.05) were observed in the CLS between T0 and T2 as well as T2 and T4; T4 was not different from T6. The ULS showed no differences between T0 and T2, while T2 was different from T4 and T4 was different from T6 (*p* < 0.05). The RR for the ULS at T2 was 23.8 (95% CI 3.45–164.18; *p* < 0.05), and the RR for the ULS at T4 was 2.58 (95% CI 1.67–3.99; *p* < 0.05).

## 4. Discussion

The patients selected for this study showed, at time 0, a heterogeneous group of lesions. The muscular and tendinous components of the Biceps Brachii, the Supraspinatus and the Infraspinatus, alone or in combination, were injured. This is not surprising, considering the intense workload that the teno-muscular shoulder structures have to sustain during a jump in agility trials [11].

According to human researches, tendons can carry loads and maintain tension for a long time since their oxygen consumption is 7.5 times lower than that of the skeletal muscles, and the metabolism of those structures is primarily anaerobic [21]. According to research involving humans, tendon healing after injuries has three phases. The first phase (inflammatory phase) lasts 24 h and phagocytosis of the necrotic materials predominates; however, at the same time, type III collagen synthesis starts. Phase two (proliferative phase) coincides with the peak of the synthesis of type III collagen; it starts after a few days and lasts for a few weeks. Phase three (remodeling phase) starts six weeks after the trauma and is divided into two sub-phases: consolidation and maturation. In the consolidation sub-phase, the tissue undergoing repair changes from cellular to fibrous, and the collagen fibers become aligned in the direction of the stress. The maturation sub-phase starts approximately ten weeks after the original trauma and shows a gradual change from fibrous tissue to scar-like tendon tissue and subsequent remodeling, which can last for approximately one year [21,22].

Three phases were described in the human literature for muscle healing, the first two being short. The destruction phase takes one or two days, and it is characterised by an acute inflammatory response with extreme cell reactions. The repair phase begins three to five days after the destruction phase and corresponds to the beginning of myofibre production. It is completed in four weeks. The remodeling phase includes the maturation of the regenerated myofibres and reorganisation of the scar tissue, which leads to the recovery of the functional activity of the muscle. It can take a long time to complete since much time is needed before the strength of the muscle is restored to the pre-injury level [23,24,25].

The veterinary literature about tendon healing in the dog is scarce. Still, many papers were written on superficial digital flexor tendon healing of the horse, showing similarity with the phases of human recovery [26,27]. Three phases of tendon healing were described in the human and equine literature: the first inflammatory phase starts 24 h after the injury and lasts a few days, the second proliferative phase that starts subsequently and lasts about six weeks and the last remodeling phase that can be divided into the consolidation and maturation phase. Those phases last from 10 weeks to one year [21].

Based on these data and assuming that the characteristics of equine digital flexor tendon are similar to those of the dog, it was decided to recheck the patients using ultrasound after two, four and six months to be able to assess the consolidation phase and the early and middle parts of the maturation phase of the tendons coupled with the long-lasting muscle remodeling phase.

Scientific evidence regarding the efficacy of canine physiotherapy and rehabilitation after an injury is scarce; randomised controlled trials and meta-analyses regarding the application of specific therapeutic modalities are lacking. A guide to approaching the rehabilitation of canine shoulder conditions was published in 2015, and the authors stressed the need for accurate identification of the anatomical structure involved in the lesion [4,28].

The first goal in the acute inflammatory phase was to reduce swelling and pain and increase the speed of the healing process. In the cases which showed fluid accumulation (articular or peritendinous) without tendon or muscle structural lesions, diathermy was associated with Low-Level Laser Therapy (LLLT), set in non-thermal modality, to enhance the anti-edemigen therapeutic effect [29]. Therapeutic ultrasound was used in pulsed mode to improve collagen organisation without an excess of the proinflammatory effect [15]. Low-Level Laser Therapy was chosen in place of TUS in cases of fluid accumulation without tendon or muscle structural lesions due to its effects which lead to neo-angiogenesis and pain control [30]. Hydrotherapy has a positive effect on active Range of Motion (ROM), muscular strength and blood circulation without overloading the injured tissues. In the present study, hydrotherapy was started as soon as possible to help prevent adhesions between the injured tissues, build up muscle mass and prevent postural damage [31,32,33].

At the two-month follow-up, in the middle of the consolidation phase, 78% of the patients were considered clinically sound on physical examination and not lame when walking and trotting and during their daily routine activity; however, none of these patients was ultrasonographically healed.

The present statistical evaluation showed that, while at T0, all the lame patients had significant lameness associated with a relevant ULS, at T2, the clinical examination underestimated the grade of the ULS in the majority of patients. Relative Risk indicated that, two months after injury, ultrasound (ULS) was 23.8 times more effective in identifying a shoulder lesion as compared to clinical lameness score (CLS). The most likely reason was that the patients were under restricted physical activity, and they were not performing sports during the time of the study. This was necessary for the healing tendinous and muscle phases and to avoid lesion relapse.

Five dogs left the study after the first follow-up (5/32; 16%), and three of the five (3/5; 60%) had a poor ultrasonographic and clinical outcome at T2. One dog was bitten by another dog and developed a tarsal fracture. One dog left the study due to the owner’s time limitation despite good improvement in the lesions. These findings suggested that the ultrasonographic aspect of the tendon could be correlated to the clinical behaviour of the lesion. A consistent relationship between the sonographic and the histological findings during tendon injury healing was demonstrated in performance horses and in human medicine [34,35]. For ethical reasons and due to the characteristics of the population observed, histological examination of the healed tissue was not possible. For this reason, the correspondence between the ultrasonographic appearance of the healing tendon and its real recovery status has yet to be proven, even if the results are promising.

The US follow-up at T4 was carried out in the first week of the maturation part of the remodelling phase of both the tendon and the muscle lesions. All the dogs that remained in the study were considered to be sound; however, 44% of the dogs at this stage (seven graded mild and five graded moderate) had lesions potentially related to lameness, similar to dogs under a high workload. Relative Risk indicated that, four months after the injury, ultrasound was 2.53 times more efficient in identifying a shoulder lesion as compared with only clinical lameness score (CLS). Despite the absence of lameness and the nearly healed lesions, the still not perfectly aligned and packed fibres could have exposed the patients to a potential reinjury as reported in the literature regarding performance in horses and humans. (Figure 1) [34,36]. For this reason, and considering the highly demanding training necessary for competition, it was decided, based on the ultrasound results, to delay the RTP of the patients for an additional six weeks. At the same time, the patient started a gradually increasing workload, intended as training and reinforcement of the mostly healed lesions and the shoulder apparatus in general.

The six-month follow-up took place during the late maturation sub-phase of the remodeling phase for both the injured tendons and the muscles. All the patients were back in training for at least two weeks. Nineteen patients (19/27; 70%) had a lesion found at US graded as minimal, for the most part, related to minor mineralisation and decreased tendon distinction with the surrounding tissues, considered to be of no clinical significance based on physical examination and the literature [37]. The patient who was still lame at T2, despite a reduced ULS score, had a ULS still graded mild at the six-month check-up. It was the only patient who was retired at twelve months due to decreased performance. A premature return to training could be the cause of poor sports performance, as suggested in the literature [38]. A second patient (1/27; 4%) was retired due to age. A third patient (1/27; 4%) was injured in the contralateral limb and retired. A fourth patient (1/27; 4%) retired from competition because, even if fully recovered, the owner was training a younger dog that appeared to perform better.

The evaluation of the dogs’ performances at the twelve-month check-up was based on owner opinion and was not objectified by force plate analysis or kinematics data. This was a limitation of the study. The absence of a control group to evaluate the effectiveness of the rehabilitation protocol was another relevant limitation. An additional limitation of the study could have been a not specific enough ULS grading. A different ULS grading or a different follow-up schedule could have improved the statistical relevance of this parameter, as suggested by the results of the study. The limited number of patients and the non-homogeneous lesions found at US were another limitation and were likely part of the cause of the partial lack of statistically significant results.

## 5. Conclusions

In conclusion, the present study suggested that the ultrasonographic appearance of tendons could be considered a reliable tool for assessing patient healing progression and that it is more accurate than physical examination. Two months between each ultrasonographic examination could be considered adequate to observe the healing progression of the tendinous and muscular lesions. The combined protocol could be used as the first step in developing a return to play decision-making flowchart.

## Figures and Tables

**Table 1:** Clinical Lameness Score (CLS).

Score	Clinical Appearance
0	No lameness
1	Lameness not perceptible at walking but perceptible at trotting
2	Lameness perceptible at walking and apparent at trotting
3	Lameness apparent at walking and severe at trotting
4	No weight bearing

**Table 2:** Ultrasound Lesion Score (ULS).

Score	Ultrasonographic Appearance
None	No lesions detected; the affected shoulder is identical to the non-affected shoulder
Minimal	The lesions were resolved but are still barely visible or
	The lesions were completely resolved, but minimal degenerative changes are present
Mild	Tendon ^+^ reduction diameter of less than 40%
	Minimal or no signs of swelling of the surrounding tissues
	No more than two structures involved ^#^
Moderate	Tendon ^+^ reduction diameter of less than 40% Swelling of the surrounding tissues of an area of less than 5 mm Maximum of three structures involved ^#^
Severe	Tendon ^+^ reduction diameter greater than 40%
	Swelling of the surrounding tissues of an area larger than 5 mm Three or more structures involved ^#^

^+^ One or more of the following tendons: biceps tendon, supraspinatus or infraspinatus. ^#^ The term “structures” is used to include all the soft tissues that show lesions, including tendons, muscles, joint capsule, tendon sheath.

**Table 3:** Dogs with (score > 1) or without (score < 1) clinical lameness using the Clinical Lameness Score (CLS) and dogs with (score mild to severe) or without (score none) lesions found at ultrasound using the Ultrasound Lesion Score (ULS) at their first visit (T0) and at two months (T2), four months (T4) and six months (T6) after T0.

Time	Lesions	CLS	ULS	*p*
T0	With	32/32; 100%	32/32; 100%	ns
	Without	0/32; 0%	0/32; 0%	ns
T2	With	7/32; 22%	32/32; 100%	<0.05
	Without	25/32; 78%	0/32; 0%	<0.05
T4	With	0/27; 0%	12/27; 44%	<0.05
	Without	7/27; 100%	15/27; 56%	<0.05
T6	With	0/27; 0%	1/27; 4%	ns
	Without	27/27; 100%	26/27; 96%	ns

**Table 4:** Relative Risk (RR) of the Ultrasonographic Lesion Score (ULS) as compared to the Clinical Lameness Score (CLS). The CLS is used as a control scoring system. N: number of patients.

Time	Scoring System According to the CLS and the ULS	N	%	RR	95% Confidence Interval	*p*
T2	CLS	32/32; 100%	32/32; 100%	23.8	3.45–166.18	<0.05
	ULS	0/32; 0%	0/32; 0%			
T4	CLS	7/32; 22%	32/32; 100%	2.51	1.67–3.99	<0.05
	ULS	25/32; 78%	0/32; 0%			
T6	CLS	0/27; 0%	12/27; 44%	1.02	0.15–4.19	ns
	ULS	7/27; 100%	15/27; 56%			

## Data Availability

All data contained within this article are available. Interested qualified researchers may request additional information.

## Supplementary Materials

The following supporting information can be downloaded at: https://www.mdpi.com/article/10.3390/ani12010078/s1, Table S1: The patient data, US results, and grading at Times 0, 2, 4 and 6 are summarised as Supplementary Materials. Clinical evaluation of the lameness is also included.
